# Comprehensive Humoral and Cellular Immune Responses to SARS-CoV-2 Variants in Diverse Chinese Population

**DOI:** 10.34133/2022/9873831

**Published:** 2022-06-16

**Authors:** Jiwei Li, Jing Wu, Qiuyue Long, Yan'an Wu, Xiaoyi Hu, Yukun He, Mingzheng Jiang, Jia Li, Lili Zhao, Shuoqi Yang, Xiaoyong Chen, Minghui Wang, Jianshi Zheng, Fangfang Wu, Ruiliang Wu, Lihong Ren, Liang Bu, Houzhao Wang, Ke Li, Lijuan Fu, Guojun Zhang, Yali Zheng, Zhancheng Gao

**Affiliations:** ^1^Department of Respiratory, Critical Care and Sleep Medicine, Xiang'an Hospital of Xiamen University, Xiamen, Fujian, China; ^2^School of Medicine, Xiamen University, Xiamen, Fujian, China; ^3^Department of Clinical Laboratory, Xiang'an Hospital of Xiamen University, Xiamen, Fujian, China; ^4^Department of Respiratory and Critical Care Medicine, Peking University People's Hospital, Beijing, China; ^5^Department of Thoracic Surgery, Xiang'an Hospital of Xiamen University, Xiamen, Fujian, China; ^6^Department of Pediatrics, Xiang'an Hospital of Xiamen University, Xiamen, Fujian, China; ^7^Department of Critical Care Medicine, Xiang'an Hospital of Xiamen University, Xiamen, Fujian, China; ^8^Department of Infectious Diseases, Xiang'an Hospital of Xiamen University, Xiamen, Fujian, China; ^9^Cancer Research Center and The Department of Breast-Thyroid-Surgery, Xiang'an Hospital of Xiamen University, Xiamen, Fujian, China

## Abstract

The SARS-CoV-2 variants have been emerging and have made great challenges to current vaccine and pandemic control strategies. It is urgent to understand the current immune status of various Chinese populations given that the preexisting immunity has been established by national vaccination or exposure to past variants. Using sera from 85 individuals (including 21 convalescents of natural infection, 15 cases which suffered a breakthrough infection after being fully vaccinated, and 49 healthy vaccinees), we showed significantly enhanced neutralizing activities against SRAS-CoV-2 variants in convalescent sera, especially those who had been fully vaccinated. The neutralizing antibodies against Omicron were detectable in 75% of convalescents and 44.9% of healthy vaccinees (*p* = 0.006), with a GMT of 289.5, 180.9-463.3, and 42.6, 31.3-59, respectively. However, the neutralizing activities were weaker in young convalescents (aged < 18 y), with a detectable rate of 50% and a GMT of 46.4 against Omicron. We also examined and found no pan-sarbecovirus neutralizing activities in vaccinated SARS-CoV-1 survivors. A booster dose could further increase the breadth and magnitude of neutralization against WT and variants of concern (VOCs) to different degrees. In addition, we showed that COVID-19-inactivated vaccines can elicit Omicron-specific T-cell responses. The positive rates of ELISpot reactions were 26.7% (4/15) and 43.8% (7/16) in the full vaccination group and the booster vaccination group, respectively, although without statistically significant difference. The neutralizing antibody titers declined while T-cell responses remain consistent over 6 months. These findings will inform the optimization of public health vaccination and intervention strategies to protect diverse populations against SARS-CoV-2 variants. *Advances*. Breakthrough infection significantly boosted neutralizing activities against SARS-CoV-2 variants as compared to booster immunization with inactivated vaccine. Vaccine-induced virus-specific T-cell immunity, on the other hand, may compensate for the shortfall. Furthermore, the public health system should target the most vulnerable group due to a poorer protective serological response in both infected and vaccinated adolescents.

## 1. Introduction

The pandemic of coronavirus disease 2019 (COVID-19) has been ongoing for over two years, creating great challenges for the public health system. Numerous genetically distinct lineages with respective mutations have evolved and have been driving recurrent waves of severe acute respiratory distress syndrome coronavirus-2 (SARS-CoV-2) infection.

The lineage B.1.1.529 (Omicron), a heavily mutated strain that harbors 15 mutations located in the receptor-binding domain (RBD) and 8 mutated residues in the N-terminal domain (NTD) relative to the wildtype (WT) virus [1], has raised serious concerns about the diminished protection conferred by preexisting immunity as soon as it occurred [2]. Multiple studies have confirmed a large reduction in neutralization titers against Omicron and the failure of many potent monoclonal antibodies to neutralize the variant [3–5]. The Omicron variant is associated with increased transmissibility, a higher viral load, longer duration of infectiousness, and high rates of breakthrough infection and reinfection, resulting in it rapidly becoming the globally dominant variant [6, 7]. However, the clinical data administered that Omicron-infected individuals have significantly reduced odds of severe disease compared with individuals infected earlier with the Delta variant [8]. Some of this reduced severity is probably a result of previous immunity elicited by exposure to past variants, vaccines, and boosters.

As mentioned above, Omicron has emerged at a time when vaccine immunity is increasing in the world. As in China, 87.8% of its population has been vaccinated against SARS-CoV-2, mostly with inactivated vaccines. A total of 1.23 billion have received the required two doses to complete vaccinations, adding that 494.4 million have received a booster shot as of January 29, 2022 [9]. However, despite the high national rate, vaccination coverage is still patchy among the elderly, children, and adolescents. Also, waning antibody titers have raised concerns about the durability of the vaccine [10]. Furthermore, although current vaccination strategies now propose the administration of a third dose, the clinical efficacy and protection against variants of concern (VOCs) remain to be determined. Therefore, it is critical to understand the comprehensive immune responses against SARS-CoV-2 variants in diverse populations.

Here, we characterized the specific humoral and cellular immunity against SARS-CoV-2 variants in different study participants. Using a pseudovirus-based neutralization assay, we assessed the cross-reactivity of neutralizing antibodies against the Wuhan-1 (WT), B.1.1.7 (Alpha), B.1.351 (Beta), B.1.617.2 (Delta), and B.1.1.529 (Omicron) variants. Additionally, we evaluated specific T-cell responses against Omicron after 2- and 3-dose vaccinations. We found that WT and VOCs are well neutralized by the serum from convalescent individuals who have been vaccinated priorly, although neutralization of Omicron was consistently lower. Booster vaccination enhanced Omicron-specific neutralization but still at significantly lower levels compared to WT. Robust T-cell responses against Omicron were observed in vaccinated healthy donors, especially those who have received a booster dose.

## 2. Results

### 2.1. Population Characteristics

Detailed information of the 85 individuals is shown in Table 1. Briefly, the Delta convalescent cases (*n* = 36) suffered COVID-19 from September 13th to September 18th, 2021, in Xiamen, and the blood samples were obtained between 15 and 40 days postinfection. Among the Delta convalescents, fifteen individuals (15/36, 38.5%) experienced subsequent breakthrough infection after two doses of inactivated virus vaccine (BBIBP-CorV, Sinopharm, Beijing CNBG, or CoronaVac, SinoVac). All the children and adolescents (8/8, 100%) in the Delta convalescent group remain unvaccinated, while nearly half of the adults (10/18, 55.6%) and elderlies (5/10, 50%) received two doses of vaccines. Among the healthy donors, 10 subjects experienced SARS-CoV-1 infection in 2003 in Beijing, who were frontline health care workers during SARS. All the healthy donors received 2 or 3 doses of vaccines except one of the SARS-CoV-1 convalescents. The blood samples were collected between 7 and 381 days after the final vaccination.

### 2.2. Reduced Cross-Neutralizing Activity to SARS-CoV-2 Variants

**Figure 1:**
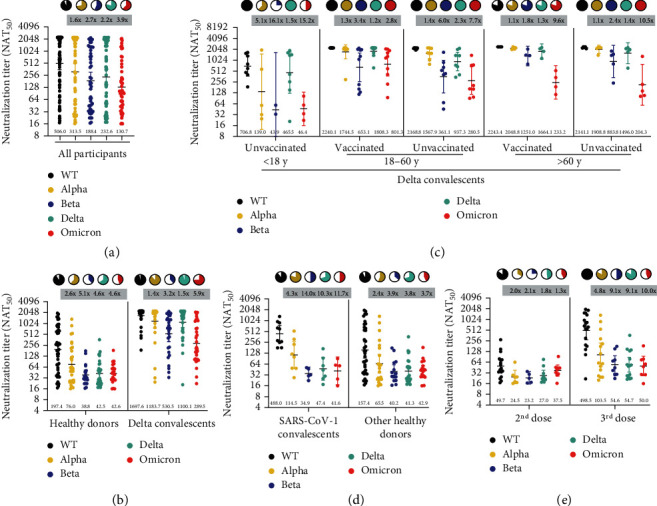
Neutralizing antibody titers against SARS-CoV-2 wild type (WT) and variants of concern (VOCs). (a) The 50% neutralization titers (NAT_50_) were determined via VSV pseudovirus neutralization assay against WT (black dots), Alpha (yellow dots), Beta (purple dots), Delta (green dots), and Omicron (red dots) variants in all samples. (b) Comparison between vaccination sera and convalescent sera. (c) Subgroup analysis of age and vaccination status in Delta convalescents. (d) Subgroup analysis of SARS-CoV-1 infection history in healthy donors. (e) Subgroup analysis of vaccination status in healthy donors. Data are presented as scatter dot plots with error bars indicating the geometric mean titers (GMT) with a 95% confidence interval (CI). The GMT values are shown at the bottom of the dots. Fold-change of GMT compared to WT by VOCs is shown at the top of each group. Pie charts show the proportion of vaccinees within each group that had detectable neutralization against the indicated SARS-CoV-2 variants. All neutralization and ELISA assays were conducted in biological duplicates.

We first determine the general neutralizing antibody (nAb) responses against WT and four VOCs. The results showed a substantial decline in both breadth and potency of nAbs (Figure 1(a)), no matter whether the nAbs were elicited by vaccination or SARS-CoV-2 infection (Figure 1(b)). Neutralization against WT was detected in 94.1% (80/85) of cases, while in 72.9% (62/85) of cases, 56.4% (48/85) of cases, 78.8% (67/85) of cases, and 62.4% (53/85) of cases when against Alpha, Beta, Delta, and Omicron variants, respectively (*p* < 0.0001). The viral neutralization titers (GMT) against the Alpha variant decreased 1.6-folds (a ratio of WT/variant) in the pseudovirus assay compared to WT (GMT 506, 95% CI 355.8-719.7), while 2.7-fold, 2.2-fold, and 3.9-fold reductions were observed in GMT against Beta, Delta, and Omicron, respectively (Supplementary Table S1). These results indicate a general reduction in neutralizing activities of sera against SARS-CoV-2 variants; the degree of decline was in the following order: Omicron > Beta > Delta > Alpha.

### 2.3. Increased Broad-Spectrum Neutralizing Antibodies after Delta Infection

We then compared the nAbs between the Delta convalescents and the healthy vaccinees (Figure 1(b)). Impressively, the Delta convalescent sera showed significantly increased neutralizing activities against WT and VOCs. The GMT of convalescent sera against WT and VOCs ranged from 1697.6 to 289.5, while the values ranged from 197.4 to 42.5 in vaccine sera, as shown in Figure 1(b) and Supplementary Table S1. The nAbs were detectable in 75%-97.2% cases of the Delta convalescents and 38.8%-91.8% participants of the healthy donors.

We further compared the neutralizing activities in different age groups of the Delta convalescents (Figure 1(c)). We observed a significant increase in the magnitude and breadth of neutralization in the adults and the elderly. However, the neutralizing abilities elicited by infection were less potent in younger cases (aged <18) when compared with the unvaccinated-infected adults (aged >18). For instance, the GMT against Omicron was only 46.4 in children and adolescents, similar to the vaccinated people (GMT 42.6).

### 2.4. Lack of Pan-Sarbecovirus Neutralizing Activity in SARS-CoV-1 Survivors

Tan et al. reported a potent cross-clade pan-sarbecovirus nAbs in SARS-CoV-1 convalescents who were immunized with the BNT162b2 mRNA vaccine [11]. Thus, we explored the differences between SARS-CoV-1 convalescents and other healthy vaccinees. All participants received 2 or 3 doses of inactivated vaccines except one of the SARS-CoV-1 convalescents. Although the GMT of the SARS-CoV-1 convalescent sera against WT is 3.1-folds higher than that of the other healthy donors, the potency of neutralization against VOCs was similar between the two subgroups, as shown in Figure 1(d). The nAbs against WT or VOCs were undetectable in the one participant who had not been vaccinated.

### 2.5. A Third Dose Slightly Increases the Breadth and Magnitude of Neutralizing Antibody Responses

As shown in Figure 1(e), the booster dose resulted in a 4.8~10-fold increase in neutralizing activity in 100% of participants against WT compared with the second vaccination. We also observed an increase in the breadth and levels of neutralizing antibodies against other variants. Eight out of 18 (44.4%) individuals who received the third dose displayed detectable serum nAbs against Omicron with a GMT of 50 (95% CI 26.6-94.1), compared with 42.8% (9/21) sera detectable with a GMT of 37.5 (95% CI 26.2-53.8) in cases who received the second doses.

**Figure 2:**
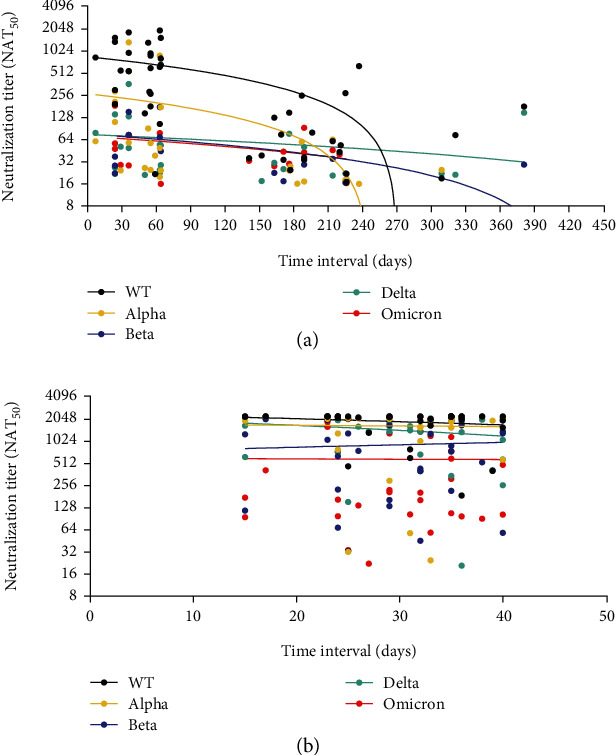
Neutralizing antibody titers decreased over time. The change of nAbs during the interval between last dose vaccination and blood sampling in healthy donors (a) and the time interval between disease onset and blood sampling in Delta convalescents (b). The nAbs show a sharp decline between 180 and 240 days across all VOCs and WT. In Delta convalescents, the nAbs slightly decreased within 30 days postinfection. The different colored dots represent nAbs against WT (black dots), Alpha (yellow dots), Beta (purple dots), Delta (green dots), and Omicron (red dots).

We also observed that the nAbs decreased over time after vaccination, as shown in Figure 2(a). A sharp decrease in serum nAbs against VOCs and WT was observed at 180-240 days post the final vaccination. The Omicron variant showed a similar decreasing pattern as the Beta and Delta variants, while the Alpha was similar to the WT. nAbs against SARS-CoV-2 variants remain consistent in the Delta convalescents within 30-40 days postinfection (Figure 2(b)).

### 2.6. Omicron-Specific T-Cell Responses in Vaccinated Healthy Donors

**Figure 3:**
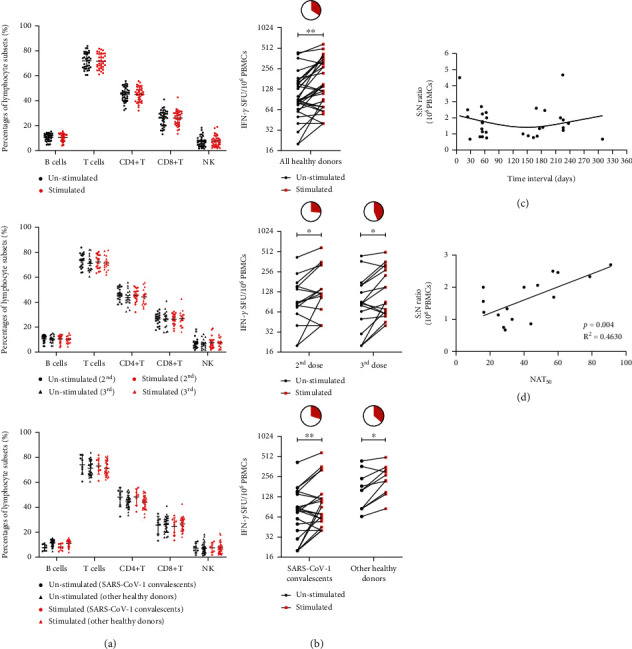
Cellular immune response to recombinant S-RBD proteins of Omicron in healthy donors. (a) The phenotypic analysis results of PBMCs from healthy donors before and after stimulation with Omicron S-RBD protein, followed by subgroup analysis of different vaccination statuses (having received the 2nd or 3rd dose of vaccination) and then the subgroup analysis between SARS-CoV-1 convalescents and other healthy donors. (b) IFN-*γ* ELISpot analysis of PBMCs from healthy donors to recombinant Omicron S-RBD proteins, followed by subgroup analysis of different vaccination statuses (having received the 2nd or 3rd dose of vaccination) and then the subgroup analysis between SARS-CoV-1 convalescents and other healthy donors. The pie charts represent corresponding proportions of positive ELISpot results within each group. (c) The signal-to-noise (S : N) ratio of SFU at different time intervals after the last dose of vaccine. (d) The correction analysis of signal-to-noise ratio and neutralizing antibody titers (NAT_50_) against Omicron S-RBD protein. ^∗^*p* < 0.05 and ^∗∗^*p* < 0.01.

We further explored whether the Omicron-specific T-cell responses could be elicited via vaccination and the possible association between nAbs and T-cell responses. First, the PBMC immune responses against Omicron were measured (Figure 3(a), Supplementary Figure S2, and Supplementary Table S2). The proportions of lymphocyte subsets remained consistent before and after stimulation of PBMCs with the Omicron-spike protein. Similarly, the subgroup analysis revealed no differences in people who received the third dose of vaccination or who survived from SARS-CoV-1 infection.

The Omicron-spike-specific T-cell responses were measured via ELISpot analysis (Figure 3(b), Supplementary Figure S3, and Supplementary Table S3). We observed that although the numbers of IFN-*γ*-secreting T cells were similar, the positive reactions (defined as the signal − to − noise ratio ≥ 2) were higher in cases who received a booster dose (7/16, 43.8%), compared to those who received a second dose (4/15, 26.7%), although without significant difference. No significant difference in T-cell reaction intensity was detected between SARS-CoV-1 convalescent and other healthy donors, with positive reaction rates of 30% (3/10) and 36.4% (8/22), respectively. Furthermore, the virus-specific immune response of T cells lasted over 6 months postvaccination (Figure 3(c)). In addition, a significant positive correlation was observed between the activation intensity of T-cell responses and NAT_50_ (*p* = 0.0037, *R*^2^ = 0.4630), as shown in Figure 3(d).

## 3. Discussion

In the current study, we characterized SARS-CoV-2-specific humoral and cellular immunity in different Chinese populations. Sera from vaccinated-infected patients revealed the most powerful cross-neutralizing abilities against SARS-CoV-2 variants, followed by sera from natural infection convalescents, sera from individuals who received the third dose of inactivated vaccine, and, last, sera from healthy donors who received a second dose of inactivated vaccine. The Omicron-specific T-cell response was observed in healthy vaccinees. The neutralizing antibody titers were positively correlated with the intensity of Omicron RBD-specific T-cell responses. The neutralizing antibody titers declined over time while T-cell responses remain consistent or even increase. In addition, the broad antibody responses were weaker in younger convalescents (aged <18), underscoring an extra focus on these vulnerable populations.

Consistent with most research, SARS-CoV-2 VOCs showed immune escape capacities from both convalescent sera and vaccine sera. However, we observed that breakthrough infection significantly boosts the serum neutralizing capacity elicited by postvaccination. A recent study also observed a robust cross-neutralization against Omicron in vaccinees that experienced breakthrough infections [12]. Likewise, vaccination can boost cross-variant neutralizing antibodies elicited by SARS-CoV-2 infection [13, 14]. The comparison between human immune sera following breakthrough infection and vaccination after natural infection showed that they can both broadly neutralize SARS-CoV-2 variants to a similar degree [15]. Together, it is assumed that an additional antigen exposure, no matter vaccination or infection, can boost broad and robust neutralization against SARS-CoV-2 variants.

We observed that the broad neutralization elicited by infection was much weaker in younger convalescents. The results closely match those obtained in previous studies, which revealed a low protective serological response in both infected and vaccinated adolescents [16, 17]. A reduced breadth of anti-SARS-CoV-2-specific antibodies was observed in children, predominantly generating IgG antibodies specific for the S protein but not the N protein [17]. These results suggest a distinct humoral immune response in children compared to adults, with implications for age-targeting vaccine implementation and effective child protection strategies.

Concerns about the efficacy of booster vaccination in the real world are rising. As shown in our study, the third dose of inactivated vaccine could only slightly improve neutralization against variants, consistent with other studies [18–21]. A study in nonhuman primates indicates that even low titers of nAbs are sufficient to prevent experimental SARS-CoV-2 infection [22]. The protection efficacy was apparent if CD8^+^ T-cell responses are mounted, indicating that T-cell immune response may ameliorate the deficiency of nAbs in defending against SARS-CoV-2 infection.

The virus-specific T-cell repertoires could be shaped following natural infection or vaccination [23–25]. Inspiringly, only 3%-7% of previously identified T-cell epitopes are affected by mutations in the various VOCs [26, 27]. The SARS-CoV-2-specific T-cell immune repertoires could still recognize the highly mutated S protein of Omicron [28]. Furthermore, T-cell responses to SARS-CoV-2 antigens remain consistent or even increase over time, whereas antibody responses wane [23, 24], consistent with our data. A positive correlation persists between the magnitude of T-cell immune response and the titer of sera neutralizing antibodies, as indicated in our and others' studies [24, 25]. Taken together, we assumed that a higher humoral immune response may induce a stronger cellular response. This may further rationalize the current booster vaccination strategy since a vaccine is much safer than getting enhanced immunity via infection, especially in people with multiple chronic conditions [29].

We indicate that breakthrough infection or booster immunization improved the neutralizing activities against VOCs, including Omicron, despite the small number of participants studied. Adults and the elderly revealed more effective humoral responses than the child and adolescents. Furthermore, vaccine recipients retain T-cell immunity to the Omicron variant, presumably compensating for the lack of neutralization in avoiding or reducing severe COVID-19 infection. The long-term adaptive immunity may be key to protection against SARS-CoV-2 variants and even future coronaviruses. Our study supports the current vaccination strategy and urges the public health system to prioritize the most vulnerable children.

## 4. Methods

### 4.1. Human Subjects

Blood samples were collected from 85 individuals, including 36 Delta convalescent patients and 49 vaccinated healthy donors (registered in the Chinese Clinical Trial Registry, ChiCTR2100054156). We enrolled convalescents of different age groups to explore possible differences, including the children and adolescents (aged <18 y), the adults (aged 18-60 y), and the elderlies (aged >60 y). The infection status was confirmed via polymerase chain reactions. The vaccination records were required as well as other demographic data. Serum and peripheral blood mononuclear cell (PBMC) samples were isolated and stored at -80°C until analysis. Briefly, blood from study participants at convalescent time points was processed in a BSL2 laboratory at Xiamen University. Serum samples were heat-inactivated at 56°C for 30 minutes before use. PBMC from all collected blood samples were isolated via Ficoll-Paque density gradient centrifugation. Study approval was obtained from the Ethics Institute of Xiamen University Xiang'an Hospital (XAHLL2021025). All participants provided written informed consent.

### 4.2. Cell Lines

HEK293T cells (Procell) were cultured in DMEM +10% fetal bovine serum (FBS) and grown at 37°C in a 5% CO_2_ setting. We cotransfected plasmids encoding angiotensin-converting enzyme 2 (pLV-ACE2-3xFLAG-IRES-puro, HedgehogBio Science and Technology Ltd.) and transmembrane serine protease 2 (pLV-TMPRSS2-GFP, Sino Biological) into 293T cells to generate a stable expressing cell line. The cells were also cultured at 37°C and 5% CO_2_. Confirmation of ACE2 and TMPRSS2 expression in 293T-ACE2-TMPRSS2 cells was done via western blot (Supplementary Figure S1).

### 4.3. SARS-CoV-2 Pseudovirus Neutralization Assay

The vesicular stomatitis virus- (VSV-) based pseudoviruses expressing the S protein of several SARS-CoV-2 variants were purchased from Vazyme Biotech, including the WT and 4 VOCs (Alpha, Beta, Delta, and Omicron). The luciferase gene was incorporated into the VSV vector and can be expressed after infection with the pseudotyped virus. The TCID_50_ (50% tissue culture infectious dose) value [30] was used to quantify virus concentration according to the manufacturer's instructions.

Neutralization assays were performed on 293T-ACE2-TMPRSS2 cells. Serum samples were 1 : 16 diluted, followed by a 3-fold serial dilution. The diluted sera (50 *μ*L) were mixed with pseudotyped SARS-CoV-2 viruses (650 TCID_50_ per well) in 96-well plates and incubated at 37°C and 5% CO_2_ for 1 h, then cocultured with the 293T-ACE2-TMPRSS2 cells for the next 24 h [31]. The chemiluminescence signals were measured in relative luminescence units (RLU) using a Bright GloTM luciferase assay system with a GloMax® Navigator Microplate Luminometer. The neutralizing titers (NAT_50_) were defined as the 50% inhibitory dilution (ID_50_) which was calculated with the highest dilution of plasma that resulted in a 50% reduction of relative light units compared with virus control. NAT_50_ below 16 was considered negative. The NAT_50_ values within groups were summarized as geometric mean neutralizing titers (GMT) with a 95% confidence interval (95% CI).

### 4.4. Flow Cytometry

The thawed PBMC was incubated overnight at 37°C and 5% CO_2_ in RPMI 1640+10% FBS. The next day, cells were harvested and then seeded at 2 × 10^5^ cells per well in 24-well plates. For either assay, 10 *μ*g/mL recombinant SARS-CoV-2 omicron spike RBD protein (Sino Biological) was added to the experimental well while the DMSO in PBS was added to the negative control well. The cells were incubated for 36 h at 37°C and 5% CO_2_ before flow cytometry analysis. PBMC were surface stained with fluorescently labeled antibodies to CD3 (FITC), CD19 (PE/Cyanine7), CD4 (PerCP/Cyanine5.5), CD8a (APC), CD56 (BV421), and CD16 (PE) in the dark at 4°C for 30 min. Subsequently, the cells were washed with PBS and stained with Zombie dye (NIR) in the dark at room temperature for 10 min. All FACS antibodies were purchased from BioLegend. After being washed and resuspended, the samples were analyzed using a CytoFlex S cytometer (Beckman Coulter). For each assay, 10,000 events were sampled after the exclusion of debris, doublets, and dead cells. The cellular immune responses against Omicron were measured as a percentage of CD19^+^ for B, CD3^+^ for T, CD16^+^CD56^+^ for NK, CD3^+^CD4^+^ for CD4^+^ T, or CD3^+^CD8^+^ for CD8^+^ T cells after stimulation with the spike protein.

### 4.5. Interferon Gamma (IFN-*γ*) ELISpot Assay

IFN-*γ*-secreting T cells were detected using a commercial Human IFN-*γ* precoated ELISpot kit (Dakewe) according to the manufacturer's instructions. Briefly, for either assay, approximately 1 × 10^5^ PBMCs per well were plated into 96-well ELISpot plates, then incubated in the presence of Omicron-spike-RBD protein (10 *μ*g/mL) (experimental wells), phytohemagglutinin (PHA, 5 *μ*g/mL) (positive controls), or DMSO (negative controls) for 36 h at 37°C and 5% CO_2_. The cells were subsequently lysed with 4°C deionized water and washed 5 times with PBS. Following the wash, 100 *μ*L biotinylated antibody (1 : 500) and 100 *μ*L streptavidin-HRP antibody (1 : 500) were added to each well, and the mixture was incubated at 37°C for 1 h. Then, the plate was washed and 100 *μ*L/well AEC color developing solution was added. The color reaction was developed for 20 min at room temperature in the dark and stopped by adding 200 *μ*L/well of deionized water. Finally, the spot-forming units (SFU), which indicate Omicron-spike-RBD-specific T cells, were counted using an automatic ELISpot reader. The results were considered positive if experimental wells were no less than twice the negative controls (the signal − to − noise ratio ≥ 2) [32].

### 4.6. Statistical Analysis

Statistical analyses were performed using GraphPad Prism 8.0.2 and SPSS 26.0. Flow cytometry data were analyzed using FlowJo 10.4.0. The Pearson *χ*^2^ test or Fisher's exact test was performed for a two-group analysis. One-way ANOVA with Tukey's multiple comparison test was used to compare differences among multiple groups. Statistical significance was defined as *p* < 0.05. Error bars throughout all figures represent a 95% confidence interval or one standard deviation where indicated.

## Figures and Tables

**Table 1:** Characteristics of enrolled cases.

	Healthy donors		Delta convalescents (*n* = 36)
	SARS-CoV-1 convalescents (*n* = 10)	Others (*n* = 39)	
Age			
<18, *n* (%)	-	-	8 (22.2%)
18-60, *n* (%)	10 (100%)	39 (100%)	18 (50%)
>60, *n* (%)	-	-	10 (27.8%)
Gender			
Female, *n* (%)	10 (100%)	15 (38.5%)	17 (47.2%)
Vaccination status			
Unvaccinated, *n* (%)	1 (10%)	-	21 (58.3%)
2 doses, *n* (%)	2 (20%)	21 (53.8%)	15 (41.7%)
3 doses, *n* (%)	7 (70%)	18 (46.2%)	-
Time interval^∗^ (days)	55 (55-55)	163 (54.5-205)	31.5 (25-35)

^∗^Time interval for healthy donors indicates the period between their last vaccination and blood sample collection. For Delta convalescents, it indicates the period between infection and blood sample collection. Data are presented as *n* (%) or median (IQR).

## Data Availability

The data that support the findings of this study are available within the article and its supplementary materials. Raw data are available from the corresponding authors on reasonable request.

## Supplementary Materials

Supplementary 1 Supplementary Figure S1: western blot analysis confirmed the expression of ACE2 and TMPRSS2 in 293T-ACE2-TMPRSS2 cells. Supplementary Figure S2: the gating strategy of flow cytometry. Supplementary Figure S3: representative ELISpot results.

Supplementary 2 Supplementary Table S1: GMT values with 95% CI in different subgroups. Supplementary Table S2: proportions of lymphocyte subsets in PBMCs from vaccines before and after stimulation. Supplementary Table S3: descriptive statistics of SFUs per 106 PBMCs in healthy donors in ELISpot experiment.
