# Corrigendum to “Comprehensive Humoral and Cellular Immune Responses to SARS-CoV-2 Variants in Diverse Chinese Population”

**DOI:** 10.34133/2022/9796409

**Published:** 2022-11-14

**Authors:** Jiwei Li, Jing Wu, Qiuyue Long, Yan'an Wu, Xiaoyi Hu, Yukun He, Mingzheng Jiang, Jia Li, Lili Zhao, Shuoqi Yang, Xiaoyong Chen, Minghui Wang, Jianshi Zheng, Fangfang Wu, Ruiliang Wu, Lihong Ren, Liang Bu, Houzhao Wang, Ke Li, Lijuan Fu, Guojun Zhang, Yali Zheng, Zhancheng Gao

**Affiliations:** ^1^Department of Respiratory, Critical Care and Sleep Medicine, Xiang'an Hospital of Xiamen University, School of Medicine, Xiamen University, Xiamen, China; ^2^School of Medicine, Xiamen University, Xiamen, Fujian, China; ^3^Department of Clinical Laboratory, Xiang'an Hospital of Xiamen University, School of Medicine, Xiamen University, Xiamen, China; ^4^Department of Respiratory and Critical Care Medicine, Peking University People's Hospital, Beijing, China; ^5^Department of Thoracic Surgery, Xiang'an Hospital of Xiamen University, School of Medicine, Xiamen University, Xiamen, China; ^6^Department of Pediatrics, Xiang'an Hospital of Xiamen University, School of Medicine, Xiamen University, Xiamen, China; ^7^Department of Critical Care Medicine, Xiang'an Hospital of Xiamen University, School of Medicine, Xiamen University, Xiamen, China; ^8^Department of Infectious Diseases, Xiang'an Hospital of Xiamen University, School of Medicine, Xiamen University, Xiamen, China; ^9^Cancer Research Center and the Department of Breast-Thyroid-Surgery, Xiang'an Hospital of Xiamen University, School of Medicine, Xiamen University, Xiamen, China

In the article titled “Comprehensive Humoral and Cellular Immune Responses to SARS-CoV-2 Variants in Diverse Chinese Population” [1], the location of some of the institutions in the affiliation list was incorrect. The affiliations have been updated and the corrected affiliations have been listed above.

## References

[1] Li J., Wu J., Long Q. (2022). Comprehensive Humoral and Cellular Immune Responses to SARS-CoV-2 Variants in Diverse Chinese Population. *Research*.

