# Supplementary figures and images for: Comprehensive Humoral and Cellular Immune Responses to SARS-CoV-2 Variants in Diverse Chinese Population
Source: Research (Wash D C). 2022 Jun 16;2022:9873831. doi: 10.34133/2022/9873831 (PMC9275105; doi:10.34133/2022/9873831)

**293T      293T-ACE2-TMPRSS2**

---

**ACE2**

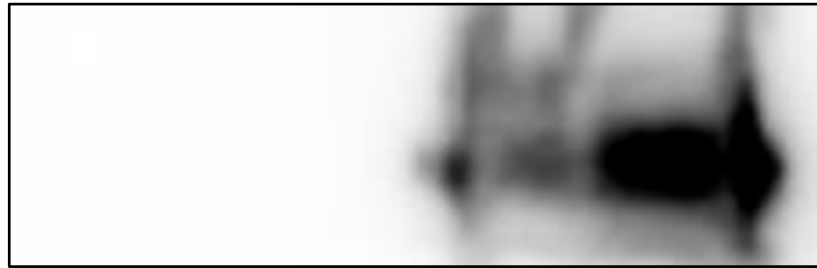

**TMPRSS2**

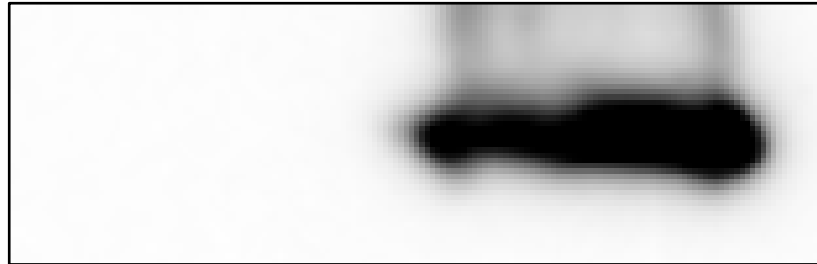

**β-actin**

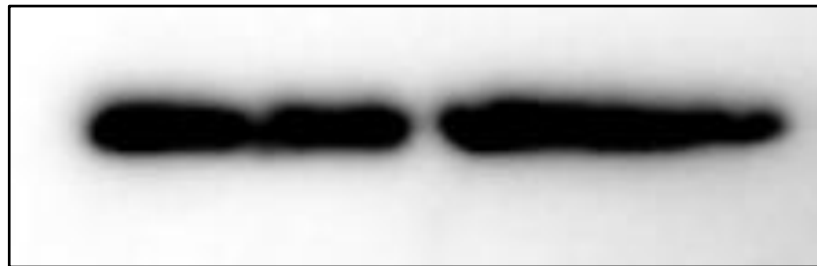

Supplement: Supplementary 1 — Supplementary Figure S1: western blot analysis confirmed the expression of ACE2 and TMPRSS2 in 293T-ACE2-TMPRSS2 cells. Supplementary Figure S2: the gating strategy of flow cytometry. Supplementary Figure S3: representative ELISpot results. [file 9873831.f1.zip › Li,Wu,Long,Wu,Hu_Figure S1.pdf]

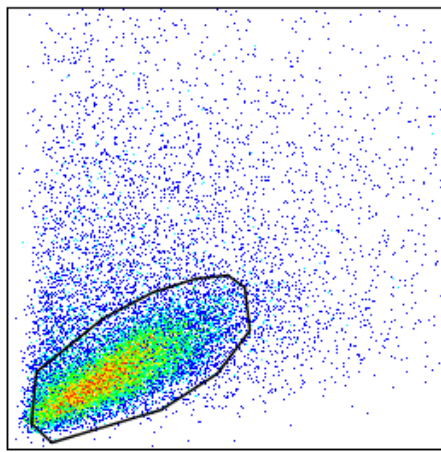

SSC-H  
FSC-H

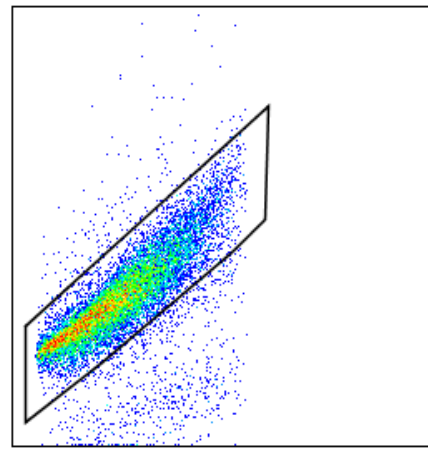

FSC-A  
FSC-H

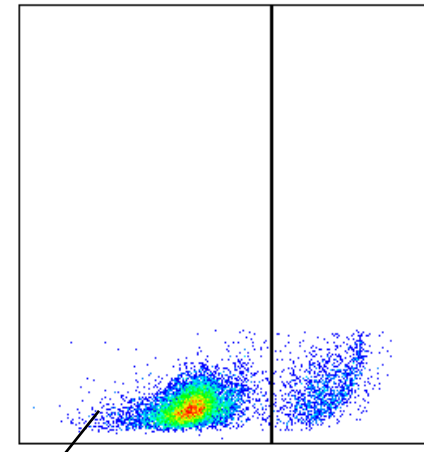

SSC-H  
Viability  
Zombie NIR

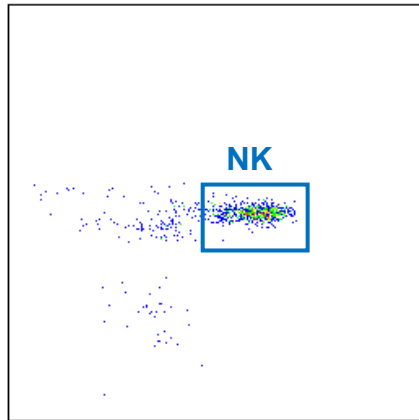

CD56  
BV421  
CD16  
PE

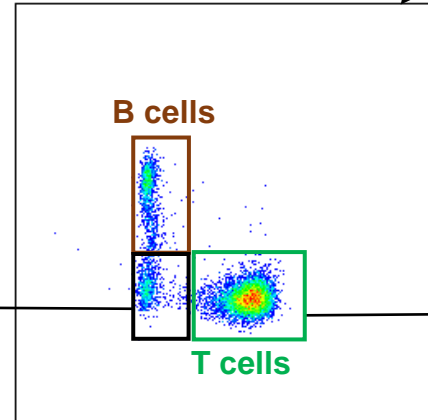

CD19  
PE-Cy7  
CD3  
FITC

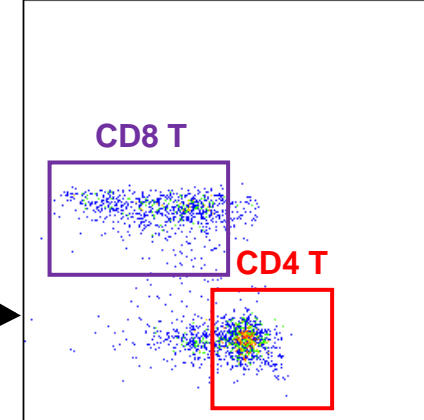

CD8a  
APC  
CD4  
PerCP/Cyanine5.5

Supplement: Supplementary 1 — Supplementary Figure S1: western blot analysis confirmed the expression of ACE2 and TMPRSS2 in 293T-ACE2-TMPRSS2 cells. Supplementary Figure S2: the gating strategy of flow cytometry. Supplementary Figure S3: representative ELISpot results. [file 9873831.f1.zip › Li,Wu,Long,Wu,Hu_Figure S2.pdf]

**HD 11**

**HD 8**

**HD 43**

**HD 46**

**HD 16**

**HD 7**

**DMSO**

15

9

13

10

17

6

**Omicron  
S-RBD**

39

42

32

27

35

15

**PHA**

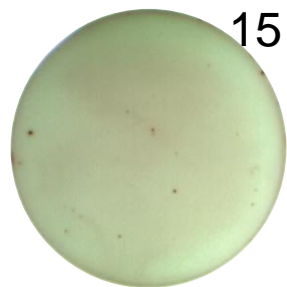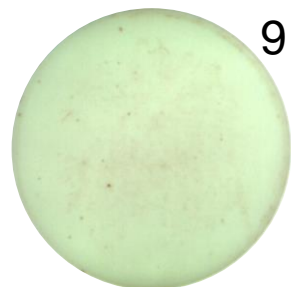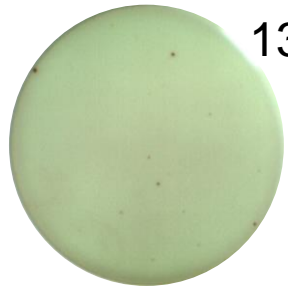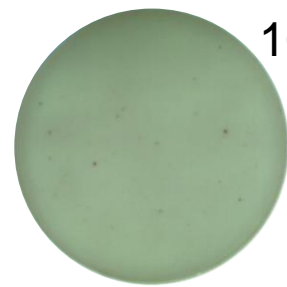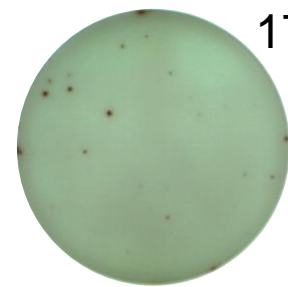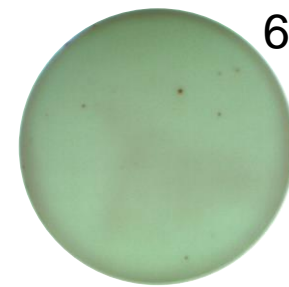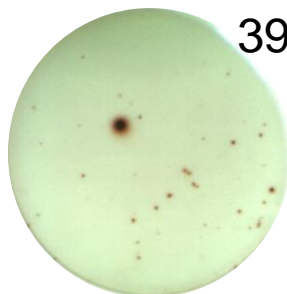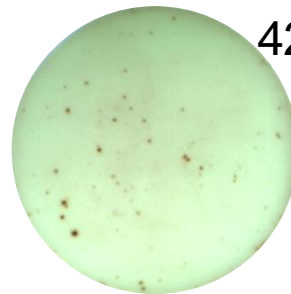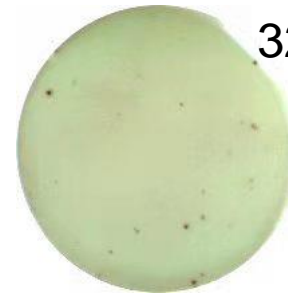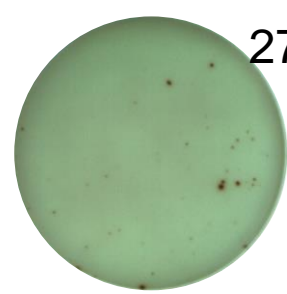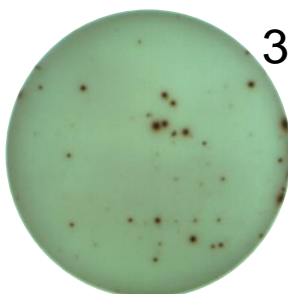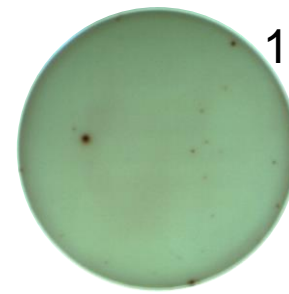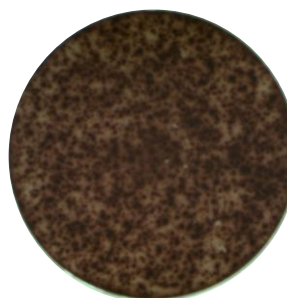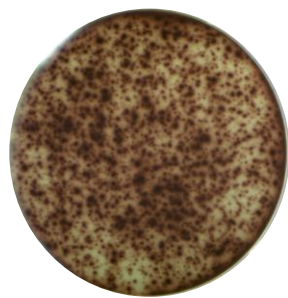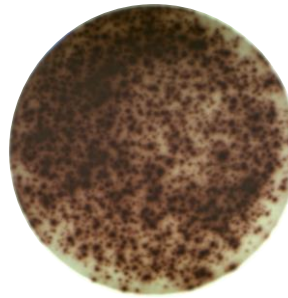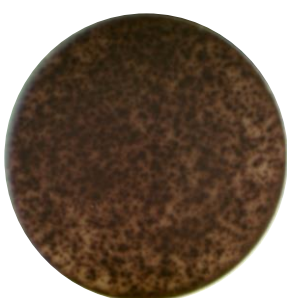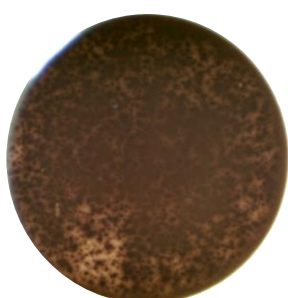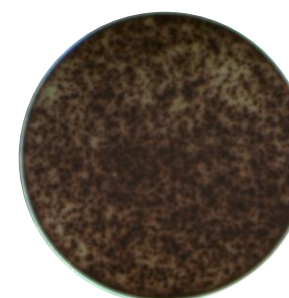

Supplement: Supplementary 1 — Supplementary Figure S1: western blot analysis confirmed the expression of ACE2 and TMPRSS2 in 293T-ACE2-TMPRSS2 cells. Supplementary Figure S2: the gating strategy of flow cytometry. Supplementary Figure S3: representative ELISpot results. [file 9873831.f1.zip › Li,Wu,Long,Wu,Hu_Figure S3.pdf]
