# Supplementary material for: Comprehensive Humoral and Cellular Immune Responses to SARS-CoV-2 Variants in Diverse Chinese Population
Source: Research (Wash D C). 2022 Jun 16;2022:9873831. doi: 10.34133/2022/9873831 (PMC9275105; doi:10.34133/2022/9873831)
Supplement: Supplementary 2 — Supplementary Table S1: GMT values with 95% CI in different subgroups. Supplementary Table S2: proportions of lymphocyte subsets in PBMCs from vaccines before and after stimulation. Supplementary Table S3: descriptive statistics of SFUs per 106 PBMCs in healthy donors in ELISpot experiment. [file 9873831.f2.doc]

|  | **All participants**  (n = 85 ) | **Delta convalescents** (n = 36) | | | | | | | | **Healthy donors** (n = 49) | | | | |
| --- | --- | --- | --- | --- | --- | --- | --- | --- | --- | --- | --- | --- | --- | --- |
|  |  | All convalescents | <18y | 18-60y (n = 18) | | | >60y (n=10) | | | All healthy donors  (n = 49) | SARS-CoV-1 convalescents (n = 10) | Other  Healthy donors (n = 39) | | |
|  |  |  | unvaccinated  (n = 8) | All 18-60y (n = 18) | Vaccinated (n = 10) | Unvaccinated (n=8) | All > 60y (n = 10) | Vaccinated (n = 5) | Unvaccinated (n = 5) |  |  | All | 2^nd^ dose (n = 21) | 3^rd^ dose  (n = 18) |
| WT | 506  (355.8-719.7) | 1697.6  (1383-2083) | 706.8  (373.8-1337） | 2208.2  (2168-2250) | 2240.1  (2231-2250) | 2168.8  (2077-2265) | 2186  (2110-2264) | 2243.4  (2240-2246) | 2141.1  (2001-2291) | 197.4  (93.9-263.8) | 488  (283.7-839.5) | 157.4  (93.9-263.8) | 49.7  (34.3-72) | 498.5  (275.3-902.8) |
| Alpha | 313.5  (195.5-502.7) | 1183.7  (759.2-1846) | 139  (12.6-1533) | 1663.7  (1287-2151) | 1744.5  (1118-2722) | 1567.9  (1143-2150) | 1969.8  (1743-2226) | 2048.8  (1823-2302) | 1908.8  (1472-2475) | 76  (36.1-118.8) | 114.5  (48.7-269.3) | 65.5  (36.1-118.8) | 24.5  (15.9-37.9) | 103.5  (48.2-222.5) |
| Beta | 188.4  (115.5-307.1) | 530.5  (324.2-868.1) | 43.9  (1.1-1678) | 501.9  (268.4-938.2) | 653.1  (262.9-1622) | 361.1  (127.9-1020) | 1031.4  (625.1-1702) | 1251  (670.6-2334) | 883.8  (320.6-2436) | 38.8  (26.2-61.8) | 34.9  (23-53.1) | 40.2  (26.2-61.8) | 23.2  (15.5-34.7) | 54.6  (30.9-96.7) |
| Delta | 232.6  (147.6-366.7) | 1100.1  (782.6-1546) | 465.5  (125.1-1732) | 1350.3  (984.6-1852) | 1808.3  (1353-2417) | 937.3  (520.8-1687) | 1568.5  (1130-2177) | 1664.1  (1022-2709) | 1496  (769.3-2909) | 42.5  (29.6-57.6) | 47.4  (22.9-53.1) | 41.3  (29.6-57.6) | 27  (19.1-38.1) | 54.7  (33.9-88.5) |
| Omicron | 130.7  (88.4-193.3) | 289.5  (180.9-463.3) | 46.4  (16-134.7) | 502.6  (283.3-891.7) | 801.3  (374.9-1713) | 280.5  (117.7-668.8) | 216.7  (113.2-414.9) | 233.2  (80.8-672.6) | 204.3  (56-745.2) | 42.6  (31.3-59) | 41.6  (17.2-100.8) | 42.9  (31.3-59) | 37.5  (26.2-53.8) | 50  (26.6-94.1) |

**Supplementary Table S1. GMT values with 95%CI in different subgroups**

Supplementary Table S2. Proportions of lymphocyte subsets in PBMCs from vaccines before and after stimulation.

|  | Un-stimulated | | | | | Stimulated | | | | |
| --- | --- | --- | --- | --- | --- | --- | --- | --- | --- | --- |
|  | Total B (%) | Total T (%) | CD4^+^T  (%) | CD8^+^T (%) | NK  (%) | Total B (%) | Total T (%) | CD4^+^T (%) | CD8^+^T (%) | NK  (%) |
| Healthy donors (n = 35) | 10.5 ± 3 | 72±6.5 | 45.2± 5.9 | 26.1± 6.3 | 7.1 ± 4.2 | 10.4 ± 3.1 | 71.6 ± 6 | 45 ± 5.9 | 26.1 ± 5.9 | 7.5 ± 4.4 |
| SARS-CoV-1 convalescents  (n = 8) | 7.6 ± 2.5 | 74 ± 7.6 | 48 ± 7.6 | 25.6 ± 7.1 | 7.2 ± 3.9 | 8.1 ± 2.6 | 73 ± 6.5 | 48.2 ± 6.6 | 24.5 ± 7 | 7.5 ± 3.8 |
| Other healthy donors  (n = 27) | 11.4 ± 2.6 | 71.4 ± 6.1 | 44.4 ± 5.1 | 26.3 ± 6.2 | 7.1 ± 4.4 | 11.1 ± 2.9 | 71.2 ± 5.9 | 44.1 ± 5.4 | 26.6 ± 5.5 | 7.5 ± 4.6 |
| 2^nd^ dose (n = 18) | 10.5 ± 3.3 | 73.4 ± 5.9 | 46.5 ± 5 | 26.5 ± 4.9 | 7.4 ± 4.6 | 10.6 ± 3.2 | 72.3 ± 5.9 | 45.5 ± 5.5 | 26.3 ± 4.7 | 7.6 ± 5 |
| 3^rd^ dose (n = 16) | 10.4 ± 3.2 | 71.1 ± 6.6 | 43.7 ± 6.8 | 26.5 ± 7.1 | 6.7 ± 4 | 10.2 ± 3.1 | 71.5 ± 5.8 | 44.3 ± 6.5 | 26.8 ± 6.5 | 7.4 ± 3.8 |

Data were presented as mean ± SD. Only one of the SARS-CoV-1 convalescents remained unvaccinated.

**Supplementary Table S3. Descriptive statistics of SFUs per 10^6^** PBMCs in healthy donors in ELISpot experiment.

|  | Un-stimulated | Stimulated |
| --- | --- | --- |
| Healthy donors (n = 32) | 125.9 ± 105.9 | 188.1 ± 147.9 |
| SARS-CoV-1 convalescents (n = 10) | 177 ± 124.8 | 256 ± 119.2 |
| Other healthy donors (n = 22) | 102.7 ± 89.9 | 157.3 ± 151.7 |
| 2^nd^ dose (n = 15) | 124.7 ± 98 | 198 ± 166 |
| 3^rd^ dose (n = 16) | 123.8 ± 118.4 | 177.5 ± 138.6 |

Data were presented as mean ± SD.
