# Supplementary material for: Comprehensive Humoral and Cellular Immune Responses to SARS-CoV-2 Variants in Diverse Chinese Population
Source: Research (Wash D C). 2022 Jun 16;2022:9873831. doi: 10.34133/2022/9873831 (PMC9275105; doi:10.34133/2022/9873831)
Supplement: Supplementary 1 — Supplementary Figure S1: western blot analysis confirmed the expression of ACE2 and TMPRSS2 in 293T-ACE2-TMPRSS2 cells. Supplementary Figure S2: the gating strategy of flow cytometry. Supplementary Figure S3: representative ELISpot results. [file 9873831.f1.zip › Li,Wu,Long,Wu,Hu_supplementary figure legend.doc]

**Supplementary Figure S1. Western blot analysis confirmed the expression of ACE2 and TMPRSS2** **in 293T-ACE2-TMPRSS2 cells.**

Plasmids encoding ACE2 and TMPRSS2 were co-transfected into 293T cells to generate a stable cell line (293T-ACE2-TMPRSS2 cells). β-actin was used as the internal control.

**Supplementary Figure S2. The gating strategy of Flow cytometry.**

Representative gating of CD19^+^ B cells, CD3^+^ T cells, CD4^+^ T cells, CD8^+^ T cells and CD16^+^CD56^+^ NK cells from donor PBMCs is shown. Briefly, live cells are gated as Zombie UV-. Cells were then gated as CD19^+^, CD3^+^ or CD19^-^CD3^-^ cells. T cells were further subdivided into either CD8^+^ or CD4^+^ populations. CD3^-^CD19^-^ cells were then divided into CD56^+^CD16^+^ NK cells. For each assay, 10,000 events were sampled after the exclusion of debris, doublets, and dead cells.

**Supplementary Figure S3. Representative ELISpot results.**

Representatively positive ELISpot results from different healthy donors. Approximately 1×10^5^ PBMCs per well were incubated in the presence of Omicron spike RBD protein (10μg/mL) (experimental wells), phytohemagglutinin (PHA, 5μg/mL) (positive controls), or DMSO (negative controls) for 36h at 37℃ and 5% CO_2_. The spot-forming units (SFU) were counted using an automatic ELISpot Reader and the numbers of SFU were shown at the top right of the figure.
